# Sol–Gel-Derived Ni_3_Al Coating on Nickel Alloy for Oxidation Resistance in Supercritical Water Environments

**DOI:** 10.3390/ma15196566

**Published:** 2022-09-22

**Authors:** Yuelong Pan, Zhidong Zhang, Daoyuan Wang, Hao Guo, Qiwu Shi, Tiecheng Lu

**Affiliations:** 1 China Nuclear Power Engineering Co., Ltd., Shenzhen 518124, China; 2 College of Materials Science and Engineering, Sichuan University, Chengdu 610064, China; 3 College of Physics, Sichuan University, Chengdu 610064, China

**Keywords:** supercritical water reaction, nickel alloy, Ni_3_Al coating, sol–gel, oxidation resistance

## Abstract

Although nickel-based alloys are widely used in industries due to their oxidation and corrosion resistance, the pursuit of better performance in harsh environments is still a great challenge. In this work, we developed a sol–gel method to synthesize Ni_3_Al coating on a nickel alloy, assisted by a post-annealing process, and investigated the oxidation-resistant performance. The coating thickness can be controlled by designing the deposition times, which keep the pure Ni_3_Al phase stable. In addition, the surface morphologies indicate that the coating is compact without obvious voids or cracks. Furthermore, the oxidation-resistant property of the coating was investigated by carrying out a supercritical water oxidation experiment. The crystalline structure and surface morphology of the samples before and after 72-h oxidation demonstrated the superior oxidation resistance of the coating. This work provides a convenient method to fabricate an oxidation-resistant coating on a nickel-based alloy, which would be significant for prolonging the service life of vessels under oxidation conditions, especially for supercritical water reactions.

## 1. Introduction

Supercritical Water Oxidation (SCWO) is a treatment technology that uses supercritical water as the corrosive medium and oxygen or air as the oxidant to treat organic waste. Compared with traditional treatment technologies, SCWO has the advantages of high treatment efficiency, environmental protection, and no secondary pollution. The candidate materials for equipment reactors in supercritical water environments are mainly iron-based alloys, nickel-based alloys, titanium-based alloys, etc. [1,2,3]. However, in the supercritical water environment, the reactor is in a strong oxidation and corrosion working environment, which is caused by high temperature and pressure and high solubility. Therefore, the above alloy materials are prone to oxidization and corrosion, resulting in a sharp decrease in the reactor service life. Therefore, it is particularly important to explore advanced reactor materials for the process of supercritical water oxidation [4,5,6,7].

Several functional coating layers have been developed for improving the oxidation resistance of the alloys used in SCWO. In particular, many ceramic materials have superior chemical stability compared to metal-based materials, such as Al_2_O_3_, ZrO_2_, TiO_2_, etc. [1,8]. For example, Nagae et al. investigated the corrosion performance of Al_2_O_3_ ceramics in supercritical aqueous solutions, and the results showed that high-purity Al_2_O_3_ ceramics exhibited excellent corrosion resistance [9]. Schacht et al. investigated the high-temperature and high-pressure corrosion performance of Al_2_O_3_ ceramics in supercritical acidic aqueous solutions. They indicated that Al_2_O_3_ ceramics still exhibited high stability, even when the temperatures increased to 500 °C. Therefore, ceramics including Al_2_O_3_ have been suggested for potential application in acidic and alkaline supercritical water environments [10].

The most commonly used methods for depositing ceramic coatings include plasma spraying, electroplating, thermal spraying, and powder embedding, etc. [11,12,13]. For example, Shuwei Guo et al. prepared zirconia and titanium oxide coatings on the surface of a 316L stainless steel substrate by air plasma spraying and found that the coatings inhibited the outward diffusion of metallic elements and the inward diffusion of oxygen. It could therefore improve the corrosion resistance of the substrate. It was also reported that the corrosion resistance of titanium oxide coatings was better than that of zirconia under supercritical water environments [14,15]. Nevertheless, the oxide ceramic coatings would suffer from thermal mismatch with the metal substrates. Due to the different coefficients of thermal expansion between the two, the coatings tend to peel off during cold and hot cycles [16,17,18,19]. Although an interfacial layer between the ceramic layer and the metal has been introduced to alleviate this problem, it always needs an extra deposition process, and both the interfacial layer and the surface coating should be designed carefully to match one other. Thus, it is still significant to explore the other functional layer in this regime.

The sol–gel process is regarded as a quite promising and important method, due to its low cost, easy coating of large-scale surfaces, and flexible control of film thickness, etc. [20,21,22]. It has been widely used to fabricate both inorganic and organic films or coatings [23,24,25]. Moreover, this method is available for the treatment of various substrates, such as metals, alloys, semiconductors, and even polymers. It thus provides a convenient solution for surface protection and functionalization [26,27]. In this work, we employed a sol–gel method to fabricate an oxidation-resistant layer on a nickel alloy. Here, a Ni_3_Al coating can be formed on the nickel alloy, assisted by a post-annealing process. The coatings annealed at different temperatures were designed, and the thicknesses were also controlled by the coating times. The supercritical water oxidation experiments indicated that these sol–gel-derived Ni_3_Al coatings presented a good oxidation-resistant property. It is therefore significant for preparing functional coatings for vessels working under oxidation conditions.

## 2. Experiments

**Deposition of coatings on nickel alloy by sol–gel method** [28]: The Al-containing sol was prepared as follows: 20.42 g of C_9_H_21_AlO_3_ was poured into 162 mL de-ionized (DI) water at room temperature and then heated at 85 °C using water-bathing for 30 min. Then, 1.875 mL of nitrate was added into the solution, followed by vigorous stirring for 10 h. The solution was then heated at 90 °C for 12 h, then a light-blue sol was filtered. After ageing at room temperature for 24 h, a stabilized gel was formed through sol–gel transition. This Al-containing precursor was deposited on a nickel alloy (Inconel 625, commercially available) substrate by the spin coating method. The films were then dried at approximately 90 °C for 3 min to remove the residual moisture. The film thickness can be controlled by repeating the coating process with different times. Subsequent annealing was carried out in a furnace at 550 °C for 1 h in a static atmosphere of nitrogen, in order to achieve the final coating layer.

**Characterization:** The precursor Al-containing gel was analyzed by Fourier Transfer Infrared Spectroscopy (FT-IR). The crystalline structure of the coating was investigated by X-ray diffraction (XRD) (X’ Pert, Philips), with a 4 kW monochromatic Cu Kα (λ = 0.15406 nm) radiation source. The surface morphologies were studied by field emission scanning electron microscopy (FE-SEM) (Inspect F, FEI) with an accelerating voltage of 20 kV. The elements were detected and analyzed by X-ray energy spectrum analysis (EDS). The supercritical water oxidation experiments were carried out using a man-made device at the Institute of Advanced Technology, CAS (Guangzhou). The experiment condition was 500 °C/25 MPa/72 h.

## 3. Results and Discussions

**Figure 1:**
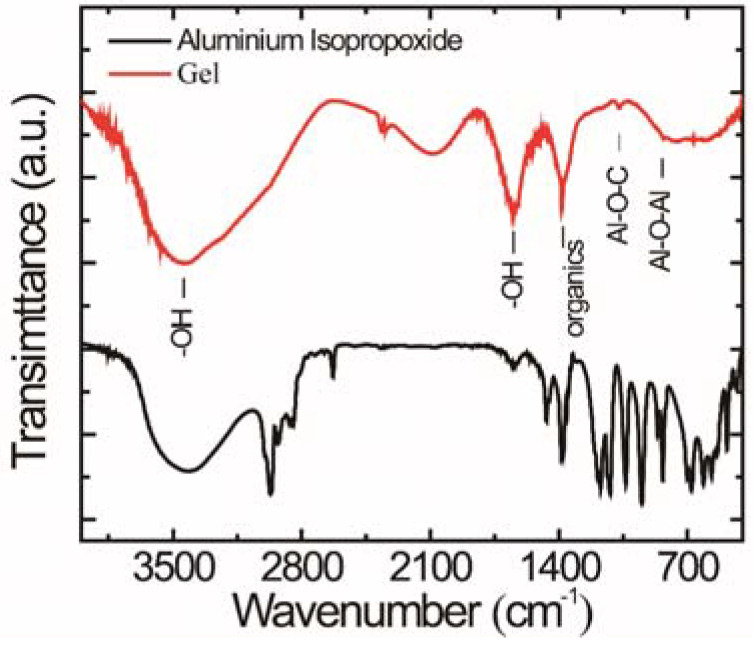
FT-IR spectra of the raw material C_9_H_21_AlO_3_ (black curve) and Al- containing gel (red curve).

The Al-containing gel is an inorganic network derived from hydrolysis and polycondensation reaction of the C_9_H_21_AlO_3_. Figure 1 shows the FT-IR spectra of the gel on KBr plates in the spectral range of 4000–400 cm^−1^. A broad peak below 850 cm^−1^ could be assigned to the characteristic distribution of Al-O-Al groups [29]. Moreover, two strong peaks at 3460 cm^−1^ and 1639 cm^−1^ can be observed, which correspond to the stretching vibration and bending vibration bands of -OH groups. They can be ascribed to water absorption and the chemical bonding of -OH groups in the gel, respectively. Furthermore, a minor peak at approximately 1075 cm^−1^ could also be detected and can be assigned to the absorption peak of Al-O-C. By contrast to the FT-IR spectrum of raw material C_9_H_21_AlO_3_, obvious changes observed in the gel can be ascribed to the hydrolysis and polycondensation reaction for the sol–gel transition.

**Figure 2:**
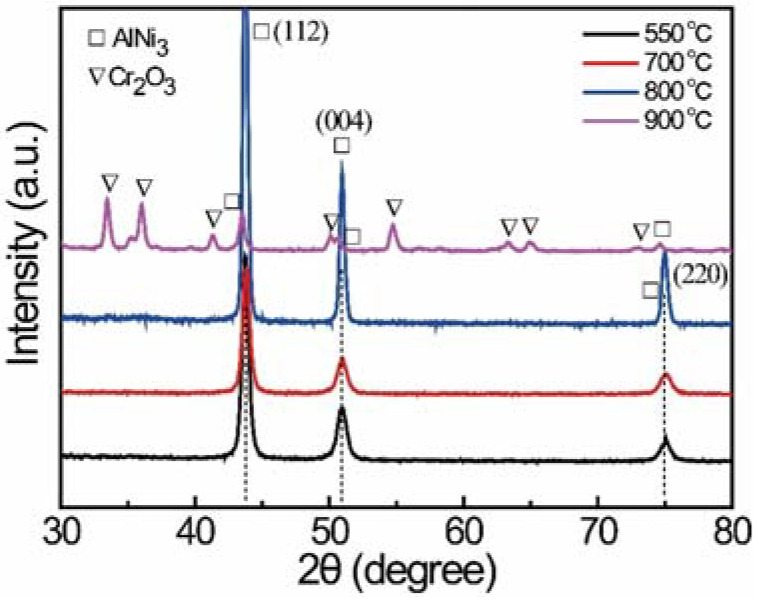
XRD patterns of the coating annealed at 550 °C, 700 °C, 800 °C, and 900 °C.

**Figure 3:**
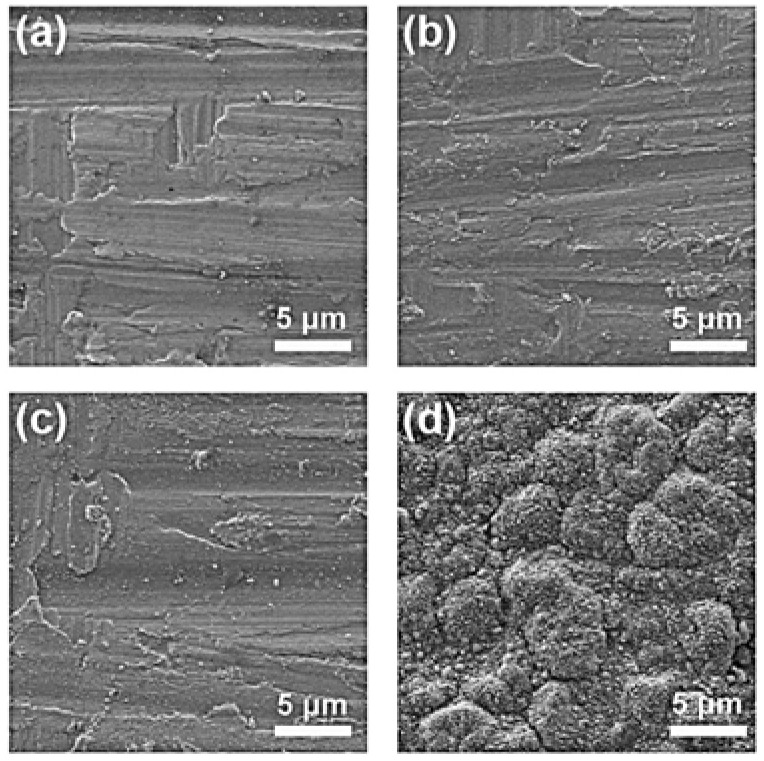
SEM morphology of the coating annealed at (**a**) 550 °C, (**b**) 700 °C, (**c**) 800 °C, and (**d**) 900 °C.

The coating was deposited on a nickel alloy substrate using a conventional spin-coating process, and then annealed at 550 °C, 700 °C, 800 °C, and 900 °C. The XRD patterns of the coatings annealed at different temperatures are shown in Figure 2. The peaks at approximately 43.7°, 50.9°, and 75.1° fit well with the diffraction peaks of the (112), (004), and (220) planes of the Ni_3_Al phase, according to PDF Card No. 50-1256. This indicates that the gel has been converted completely to Ni_3_Al at 550 °C, 700 °C, and 800 °C. However, increasing the annealing temperature at 900 °C leads to the formation of the Cr_2_O_3_ phase. It should also be noted that the annealing was performed at a static atmosphere of nitrogen; a small amount of oxygen cannot be avoided. Therefore, the formation of Cr_2_O_3_ can be explained as follows: the chromium in the nickel alloy would be oxidized but not react with aluminum at high temperature [28]. The SEM morphology of the coatings is shown in Figure 3. It also indicates that the morphology of the samples annealed at 550 °C, 700 °C, and 800 °C are quite different from that of the sample annealed at 900 °C. In particular, the surface is relatively smooth for the coating annealed at lower temperature. By contrast, the surface of the coating annealed at 900 °C is uneven and shows a strong sense of granularity, which, according to the previous XRD results, can be ascribed to the formation of an extra Cr_2_O_3_ phase. 

**Figure 4:**
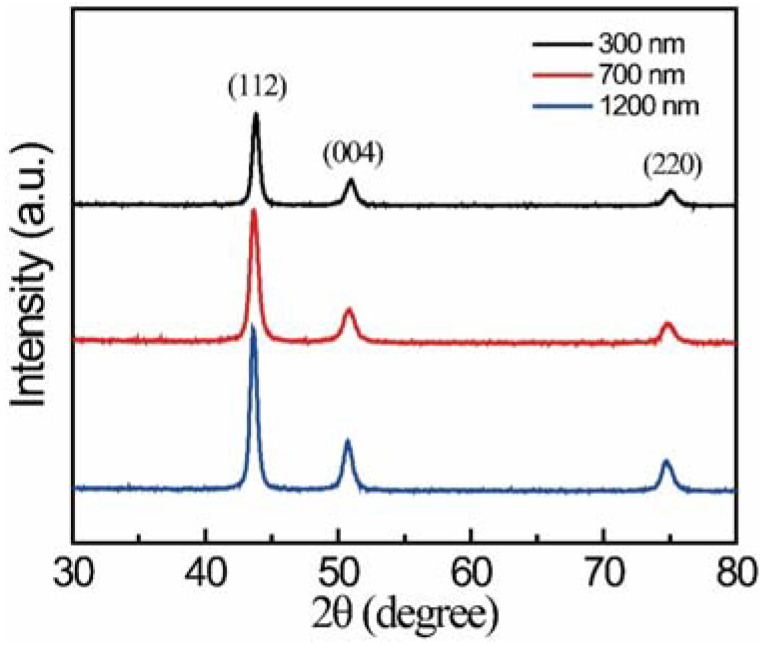
XRD patterns of the coating layer with different thicknesses annealed at 550 °C.

The precursor gel films were deposited on nickel alloy substrate and then annealed for the growth of the coating layer. Figure 4 shows the XRD patterns of the coating layers with different thicknesses. By increasing the coating thickness from 300 nm to 1200 nm, the samples indicate a stable and pure-phase of Ni_3_Al (PDF Card No. 50-1256). It is interesting that, generally, the precursor gel would transform to Al_2_O_3_ using the sol–gel method [28,30], but the Al_2_O_3_ layer deposited directly onto nickel alloy would encounter a mismatch of thermoconductivity between the oxide layer and the metal [31,32,33]. In this work, the gel layer was annealed at 550 °C, which is obviously lower than the process for fabricating Al_2_O_3_. In this case, the precursor layer would be difficult to oxidize, which is why there is no Al_2_O_3_ phase detected in the layer. It can also be ascribed to the static atmosphere of nitrogen in the annealing process. This inert atmosphere could hinder the oxidation and even play a role of reduction in this process.

**Figure 5:**
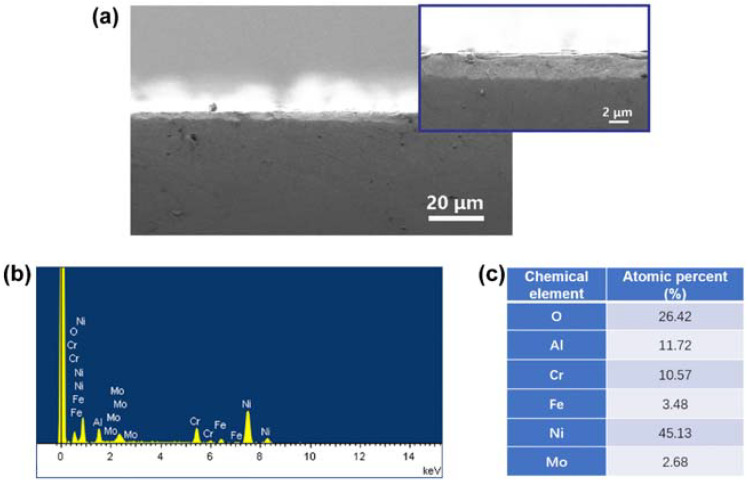
(**a**) Cross-sectional SEM morphology of the Ni_3_Al coating with a thickness of approximately 2700 nm, (**b**) EDS spectrum of the Ni_3_Al coating layer on nickel alloy, and (**c**) Atomic percent of the detected elements in the sample.

The composition of the coating layer was investigated further by analyzing the chemical elements in the layer. Figure 5a shows a typical cross-sectional SEM morphology of the coating layer. It can be seen that the coating layer is uniform, with a thickness of approximately 2700 nm. Figure 5b indicates a complicated element distribution in the samples. Combined with the XRD results in Figure 4, it suggests that most of the elements can be assigned to the matrix (only the Ni_3_Al phase can be detected on the surface of the samples). Here, the EDS measurement was carried out at a larger scale than the XRD test. This means that the elements beyond Ni and Al may be detected due to the voids from grain boundaries or cracks in the layer. Figure 5c collected the atomic percent of the elements in the sample, and the ratio of Ni/Al is ~3.85, which is larger than the ratio of Ni/Al in Ni_3_Al. This demonstrates further that part of the detected elements come from the matrix.

**Figure 6:**
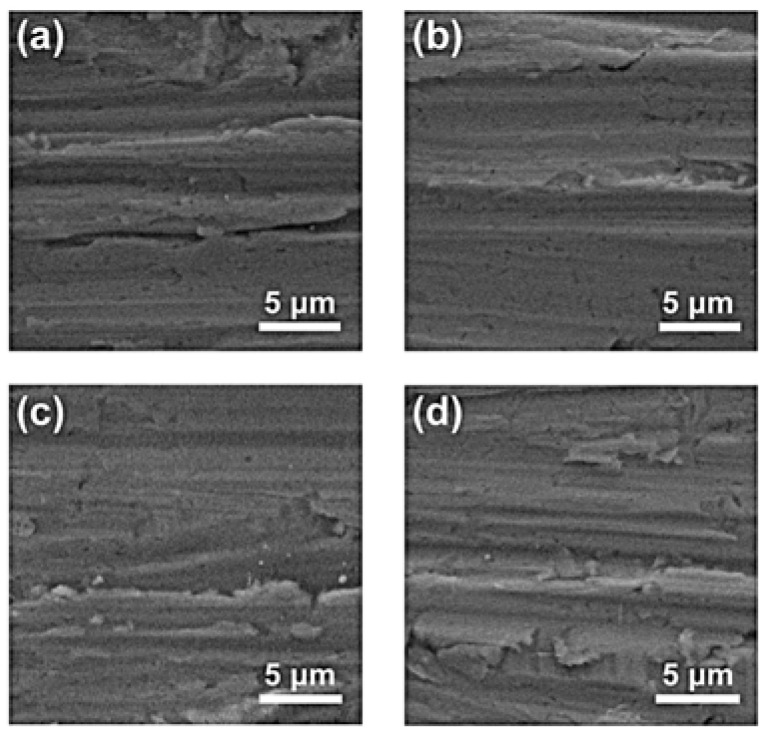
Surface morphology of the Ni_3_Al coating with thicknesses of (**a**) 300 nm, (**b**) 700 nm, (**c**) 1200 nm, and (**d**) 2700 nm.

The surface morphology of the coating layers with different thicknesses are shown in Figure 6. It can be seen that all the samples indicate a continuous surface coating layer. There are only a few micro-voids existing, which can account for the emergence of extra elements in the EDS result. However, the layer still exhibits a high quality because no crack can be found, even at a scale larger than 10 × 10 μm. Compared to Al_2_O_3_, the coefficient of thermal expansion of Ni_3_Al should be quite similar to that of nickel alloy. It could avoid the cracking of the coating layer caused by the mismatch of thermal expansivity between substrate and coating. It would thus be helpful for the stability of the coating layer at high temperatures.

**Figure 7:**
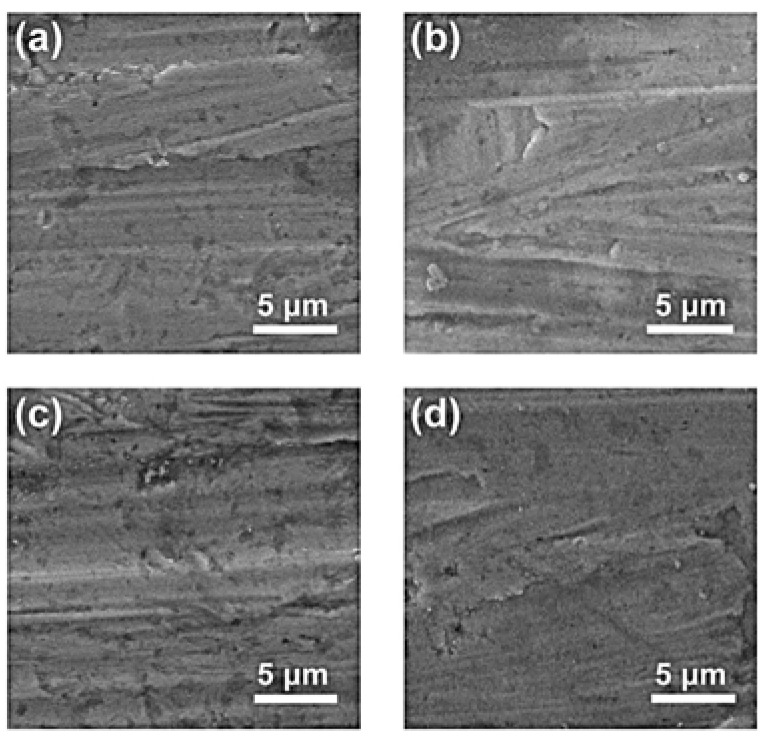
Surface morphology of the Ni_3_Al coating with different thicknesses after 72-h oxidation experiment. (**a**) 300 nm, (**b**) 700 nm, (**c**) 1200 nm, and (**d**) 2700 nm.

**Figure 8:**
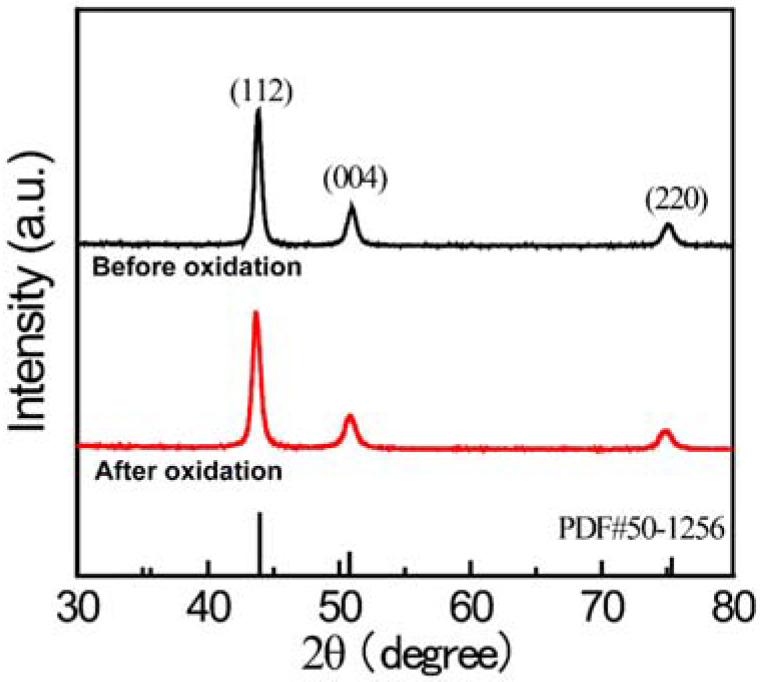
XRD patterns of the 1200-nm Ni_3_Al coating before and after 72-h supercritical water oxidation experiment.

The oxidation-resistant performance of the Ni_3_Al coating (with a thickness of approximately 1200 nm) was investigated under a rigorous supercritical water condition. The reaction temperature was set at 500 °C and the corresponding pressure reached as high as 25 MPa. Figure 7 shows the surface morphology of the coatings with different thicknesses after a 72-h oxidation experiment. Compared to the morphology showed in Figure 6, there is nearly no difference before and after the supercritical water oxidation experiment. Generally, serious cracking and even shedding would emerge in the coating layer under the rigorous supercritical water experiment [15,34,35]. However, the Ni_3_Al performs high stability, and it is remarkable that all of the Ni_3_Al coatings present an excellent oxidation-resistant property. Furthermore, Figure 8 compares the XRD patterns of the sample before and after the oxidation experiment. It can be seen that the sample keeps the pure Ni_3_Al phase in this experiment, which further proves superior oxidation resistance. It should be highlighted again that the Ni_3_Al would not encounter the mismatch of thermoconductivity between the coating layer and the alloy substrate. This is an obvious advantage compared to oxide-based oxidation resistance coating layers, such as Al_2_O_3_ and TiO_2_.

## 4. Conclusions

This work proposes a sol–gel method accompanied by annealing to deposit a Ni_3_Al coating layer on nickel alloy. The coating thickness could be controlled by repeating the coating process. By increasing the coating thickness from 300 nm to 1200 nm, the samples indicate a stable and pure-phase of Ni_3_Al. The crystalline structure, surface morphology, and EDS results indicate a high-quality coating layer. In particular, the oxidation-resistant property of the coating was investigated by carrying out a supercritical water oxidation experiment. The crystalline structure and surface morphology of the samples before and after 72-h oxidation demonstrated a superior oxidation resistance of the coating. This coating would be promising for exploring advanced coatings for supercritical water reaction vessels.

## Data Availability

The data that support the findings of this study are available from the corresponding upon reasonable request.
